# Effect of self-directed versus traditional learning model on nurses’ airway management competencies and patients’ airway-related incidents

**DOI:** 10.1186/s12912-024-02232-0

**Published:** 2024-08-27

**Authors:** Sameh Elhabashy, Amen Moawad

**Affiliations:** 1 Faculty of Nursing, Cairo University, Cairo, 11562 Egypt; 2 Aswan Heart Centre – Magdi Yacoub Foundation, Aswan, Egypt

**Keywords:** Self-directed learning, Traditional learning model, Airway management, Nurses’ competencies, Airway-related incidents

## Abstract

**Introduction:**

Self-directed learning (SDL) stands as a contemporary approach to learning, offering efficient and sustainable strategies for enhancing knowledge and practices. Given the pivotal role of nurses in ensuring patient safety and care effectiveness, this study aims to assess the impact of the SDL model compared to the traditional learning model (TLM) on elevating nurses’ airway management (AM) competencies and minimizing airway-related incidents.

**Methodology:**

The study employed an experimental research design using a posttest-only control group structure within a two-group comparison framework. Seventy-two nurses participated, with 35 in the study group and 37 in the control group at the Obstetrics and Gynecology Hospital affiliated with Cairo University, Egypt. The trial was carried out between February 2020 and July 2021. Following an assessment of SDL readiness for the intervention group, they received SDL model training based on Knowles’ SDL principles, while the control group received TLM. The primary endpoint was a significant elevation in nurses’ airway management competency, with the secondary outcome being a significant decrease in airway-related incidents reported by nurses. Competency assessments occurred immediately after completion of the intervention and again three months later.

**Results:**

A statistically significant difference was observed between the control and intervention groups regarding their practice and knowledge scores, with p-values of 0.02 and < 0.01, respectively. Additionally, the clinically relevant difference between control and intervention groups was evidenced by the effect size (ES) Cohen’s d in both practices and knowledge levels (-0.56 and − 1.55, respectively). A significant difference was also noted between the first post-assessment and the paired second post-assessment concerning nurses’ knowledge and practices among control and intervention groups, as indicated by the paired t-test with *p* < .01. Over three months, the intervention group reported 18 airway incidents, while the control group reported 24, with no statistically significant difference (> 0.05).

**Conclusion:**

The SDL model significantly enhanced nurses’ competencies in AM compared to the TLM. However, the efficacy of both learning models diminishes over time. Although nurses who underwent SDL model reported fewer airway incidents compared to those who received TLM approach of learning, no statistically significant difference was detected.

**Trial registration:**

The study has been registered with Clinical Trials.gov under the registration number (NCT04244565) on 28/01/2020.

## Background

Airway-related incidents are prevalent, critical, and intricate events that pose a significant risk to patient safety but can be avoided [1]. Airway-related incidents encompass any condition that impairs the patency of a patient’s airway, either partially or entirely. These incidents may originate from various causes, such as foreign bodies, injury, or infection [2]. The number of studies on airway-related incidents in hospitals is limited. However, it has been found that 7.04% of ICU patients experienced such incidents [3]. According to the UK National Reporting and Learning Centre, 82% of airway-related incidents occurred in patients with an endotracheal tube (ETT). Out of these incidents, 25% resulted in the patient’s death [4]. Airway management (AM) is crucial to ensuring patient safety [5]. In addition, AM encompasses nursing measures to maintain airway patency and minimize aspiration risk [6]. These actions include suctioning, oral care, basic motions such as head-tilt and chin-lift, securing the ETT, and monitoring and caring for the cuff pressure of the ETT.

Nurses play a significant role in ensuring the patient’s airways are functioning properly, particularly in emergency and critical care settings [7]. Various nursing-related malpractices can lead to airway-related incidents in patients [2, 8]. Nurses in developing countries lack knowledge and practice regarding AM [7]. According to prior research, 54.9% of nurses possess inadequate knowledge in this area [7]. Furthermore, nurses’ average knowledge score in AM was less than 50% of the highest possible score [9]. These findings demonstrate the significance of educating nurses to enhance their knowledge and practices, thereby ensuring their ability to deliver safe and qualified care. The field of nursing, including AM and other forms of care, is experiencing rapid progress due to advancements in knowledge and technology. Therefore, nurses need to be encouraged to find and utilize an appropriate learning model that can effectively achieve the concept of continuing education [10, 11].

Although nursing care has experienced significant transformations in the past thirty years, the approaches utilized in clinical training for nurses have remained unchanged [12]. The traditional learning model (TLM) in clinical teaching is a familiar and effective method of learning for nurses. It includes activities such as lectures, skills laboratory training, and supervised clinical experience. One benefit of this strategy is the opportunity to assist nurses by implementing the principles they have learned in class or skill lab to patient care. In addition, nurses feel more secure due to their awareness and familiarity [13]. Conversely, the transition from TLM to clinical instruction hinders the development of effective critical thinking skills and limits flexibility [14].

Self-directed learning (SDL) is a contemporary learning technique that provides adults with efficient and sustainable strategies for acquiring knowledge. The concept of SDL is stated in the Adult Learning Theory. This theory confirms that people are pragmatic and focused on solving problems. Their learning is mainly influenced by clinical experiences rather than passive methods [15]. SDL was conducted, and its impact on nursing students and practicing nurses who are actively involved in continuing education was assessed [16]. Moreover, it is a process in which the instructors play a facilitating role while learners actively determine their own learning needs and goals, allocate resources, and engage in self-reflection and evaluation. SDL offers various benefits, such as enhanced self-independence, confidence, sustainable learning, and autonomy [15]. Although SDL can be beneficial, there is a growing recognition that SDL is not universally applicable to all learners and circumstances. Additionally, a considerable proportion of nurses who were unfamiliar with the SDL method indicated a preference for and felt more confident with the traditional teacher-centred approach, which they commonly encountered during their postgraduate education [17].

In order to maximize the benefits, it is essential to implement SDL in a methodical manner. SDL encompasses four fundamental stages: readiness for autonomous learning, establishment of learning objectives, active participation in the learning process, and assessment of learning outcomes [18, 19]. SDL requires nurses and educators to meet specific requirements or perform certain functions. Engaging in communication about their respective perceptions is mutually advantageous for both parties. Nurses are responsible for evaluating their preparedness to gain knowledge, setting learning goals, supervising the learning process, displaying self-motivation, reevaluating and adjusting objectives as needed, and seeking guidance from the instructor when necessary. Instructors possess a range of duties, including establishing a collaborative learning atmosphere, motivating and guiding nurses in their learning endeavours, facilitating the learning process, being available for consultations when necessary, and acting as advisors rather than formal instructors [20].

In Egypt, nurses primarily participate in in-service learning activities through TLMs to improve their skills and reduce errors in practice. However, there is limited use of modern learning approaches like SDL, and there is a dearth of research on the impact and challenges of implementing SDL among Egyptian nurses. Additionally, patient care places a high emphasis on the use of AM techniques. Therefore, this study aims to assess the impact of the SDL Model in comparison to TLM on elevating nurses’ AM competencies and minimizing airway-related incidents. In order to achieve this objective, the following research hypotheses were formulated: (1) Nurses enrolled in the self-directed learning model (µ1) demonstrate higher scores in airway management practices compared to those adopting TLMs (µ2), H1: µ1 > µ2. (2) Nurses enrolled in the SDL model (µ1) exhibit higher scores in airway management knowledge than their counterparts adopting TLMs (µ2), H1: µ1 > µ2. (3) Intensive care units wherein nurses are enrolled in the SDL model (µ1) are projected to have a lower occurrence of airway-related incidents among patients compared to units utilizing TLMs (µ2), H1: µ1 < µ2.

## Theoretical framework

**Fig. 1:**
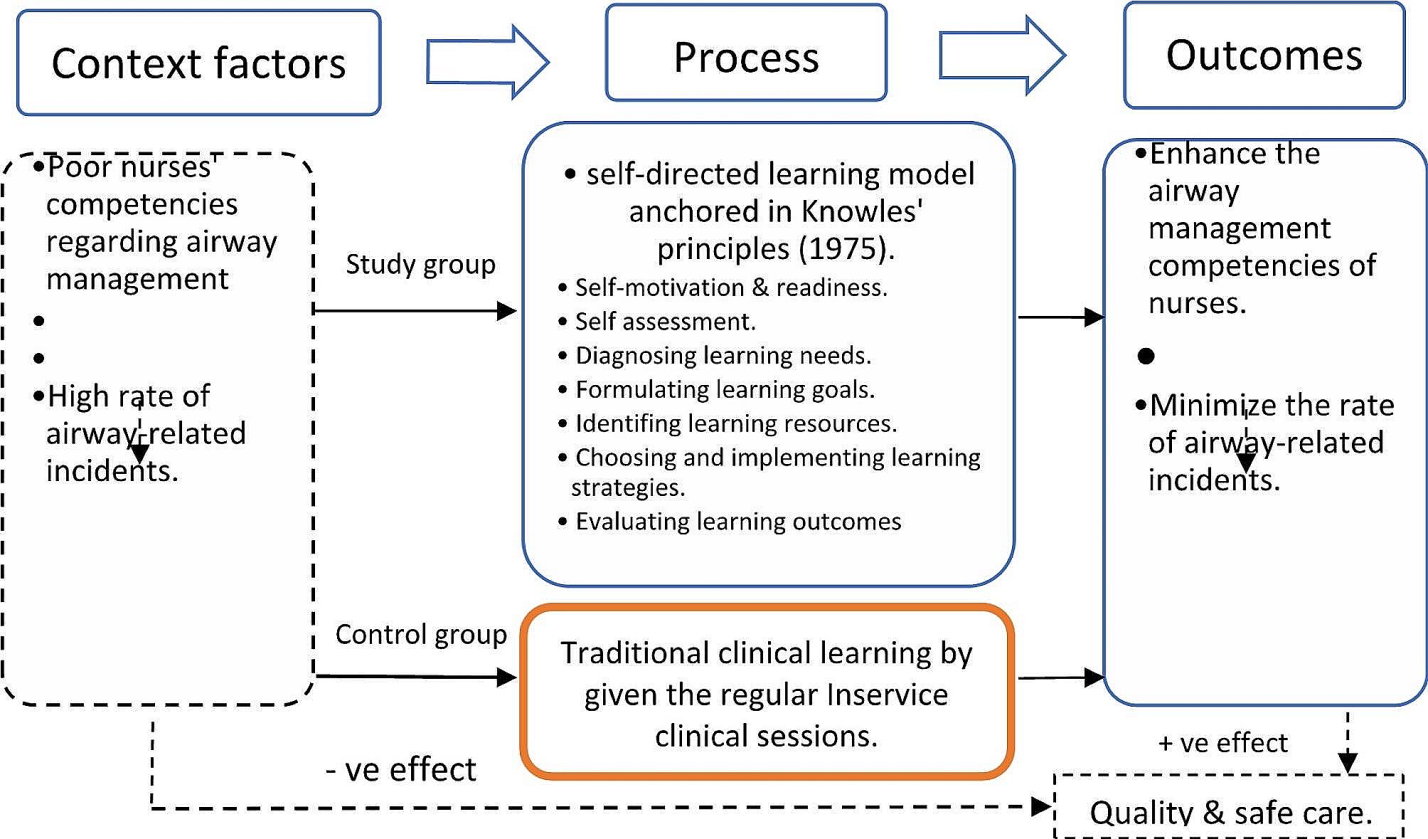
A proposed conceptual framework

Due to the correlation between inadequate nurses’ knowledge and skills regarding AM [7, 9] and the significant increase in airway-related incidents [21], it is necessary to improve nurses’ competencies through a contemporary and sustainable approach to learning. In the current study, we adopted the SDL model based on Knowles’ principles (1975) [22](Fig. 1) to assess its application effect on nurses’ competencies compared to the TLM. We aimed to address contextual factors that improve the safety and quality of care.

## Methods

### Trial design

The current study utilized a prospective open-label parallel 1:1 experimental research design employing a posttest-only control group structure within a two-group comparison framework. Pretest was not implemented to avoid a testing threat. The study was carried out between February 2020 and July 2021 and adhered to the guidelines outlined in the Consolidated Standards of Reporting Trials (CONSORT). In addition, the study was registered with Clinical Trials.gov (Registration # NCT04244565) on 28/01/2020, and Official IRB approval for the study’s execution was obtained at the Faculty of Nursing, Cairo University (CU), Egypt (approval # 2020-52). The study’s objectives, methods, potential risks, and benefits were thoroughly explained to the participants, allowing nurses sufficient review time. Participation was voluntary and had no impact on the participants’ performance appraisal. Moreover, participants had the right to withdraw from the study at any time without any repercussions on their professional evaluations. Withdrawn participants received equivalent treatment to those who remained in the regular study. Data was encoded to guarantee the anonymity and confidentiality of the subjects. It should be noted that the data collected were exclusively utilized for the specified research purpose and were not repurposed for any other purposes.

### Participants sample and setting

The research was carried out at the Obstetrics and Gynecology Hospital affiliated with Cairo University, Egypt. The architectural construction of selected units consists of pairs of sections, with each pair comprising two sections. These two sections within each pair have comparable numbers of beds, patient flow, equipment, and working nurses. Each pair was divided into two sections, with one section assigned to the control group and the other to the intervention group. This method ensured that the groups were comparable and reduced the influence of potential confounding variables related to the setting. A total of 72 voluntary participants were recruited for study inclusion, all of whom were included in the final analysis. The sample size was determined using G* Power software V.3.1.9.4 (Psychonomic Society, Madison, Wisconsin, USA) with independent t-tests, α = 0.05, Power (1-β) = 0.80, balanced allocation ratio 1:1, and Effect size = 0.65 which is consistent with the effect sizes reported in previous studies [23, 24]. The participants were divided into 35 in the study group and 37 in the control group. All selected nurses met the following inclusion criteria: hold their current position for at least one month and have a minimum of two years of critical care experience. Nurses who had plans to resign within the next six months or have been involved in any educational programs related to AM in the past six months were excluded.

### Randomization and allocation

The eligible nurses were randomly selected using a simple random sample method utilizing the computer-based program Statistical Package for Social Sciences (SPSS) V.23.0 (IBM, New York) after obtaining the sampling frame, including the pool of eligible participants. Subsequently, a sequential, random allocation was carried out for both the intervention and control groups. In order to prevent contamination between the intervention and control groups, measures were taken to ensure that each group operated in distinct sections throughout the study period. In addition, the process of randomization and allocation was carried out by an uninvolved third party.

### Outcomes

The Primary endpoint: The primary outcome was a significant elevation in nurses’ airway management competency indicated by nurses’ knowledge and practices, measured by the Airway Management Structured Questionnaire (AMSQ) and Airway Management Structured Observational Checklist (AMSOC) at the endpoint-2nd post-assessment (3 months after receiving SDL).

The secondary endpoint: The secondary outcome was a significant decrease in airway-related incidents reported during the three-month duration of patient care after receiving the SDL. The incidents were measured using Patient Safety Incident Reports (PSIR).

### Measurement tools

1. The Self-directed Learning Readiness Scale (SDLRS) was developed by Guglielmino in 1977 [25]. It is a widely used self-reported tool [26] designed to measure individuals’ perceptions of their ability and readiness to participate in SDL. It consists of 58 items, divided into eight factors. Participants respond to each item using a 5-point Likert scale, with scores ranging from “1 = almost never true of me” to “5 = almost always true of me.” The overall score varies from 58 to 290, with higher values reflecting a higher perceived level of preparedness for SDL. Individuals with a score above 226 are deemed to be above average and prepared for SDL [27]. The scale demonstrates satisfactory internal consistency, as evidenced by a Cronbach’s Alpha ranging from 0.71 to 0.88 [28]. Furthermore, the scale collects demographic data, including participant sex, years of experience, staff category, and educational level. We chose the SDLRS for our study because it uses simple and straightforward language, making it easier for participants with limited English skills to understand. Additionally, we provide clear instructions in Arabic and on-demand assistance, enhancing the participants’ sense of ease and guaranteeing easy access to the tool. Finally, this tool is in the public domain, thus necessitating no permission.
2. The Airway Management Nurses’ Knowledge Questionnaire (AMNKQ) is a tool that was developed by reviewing previous research [7, 29]. It is a self-reported tool that was used to assess nurses’ knowledge regarding AM. The questionnaire comprises 20 multiple-choice questions, with options for true and false. Each correct answer is given one point, while an incorrect answer receives zero points. Each student’s scores were summed to calculate the total score, with a maximum possible score of 20.
3. The Airway Management Nurses’ Practices Checklist (AMNPC) is a checklist designed to monitor the nurses’ practices regarding AM-related nursing practices. A third-party evaluator collected the data, we selected a team of experienced third-party evaluators who underwent comprehensive training to familiarise themselves with the (AMNPC). The training included a detailed overview of the tool, scoring criteria, and case studies. Following the initial training, raters participated in calibration sessions, which involved practice evaluations using standardised video recordings and consensus discussions to align their scoring interpretations. Also, ongoing calibration sessions and periodic reviews were conducted to maintain consistency. The tool consists of 150 steps. Those who successfully and accurately complete a step will receive a score of 2. Those who did complete a step received a score of 1. Those who incorrectly completed a step received a score of zero. The scores of each nurse were aggregated for interpretation, with the maximum score being 300.
4. The Patient Safety Incident Reports (PSIR): It is an adopted tool developed by the UK National Patient Safety Agency in 2019 for assessing airway-related incidents reported by nurses [30]. Specifically, it focuses on events associated with airway obstruction, injury, or aspiration. The data collected pertains to the type of incident, its causes, and the frequency of occurrence. A third-party evaluator regularly asked the participants to document any airway-related incidents encountered.

### Reliability and validity

Content and scope validity for the developed second and third tools were determined utilizing the Lawshe method [31]. The tool was reviewed by a panel of five experts in medicine and nursing. After calculating each item’s content validity ratio (CVR), AMNKQ and AMNPC content validity index (CVI) were 0.94 and 0.95, respectively. Before the main study, a pre-test pilot study was conducted with 12 nurses from the same setting to assess the feasibility, acceptability, and internal consistency of the tools. Internal consistency (Cronbach’s alpha) was measured to evaluate the scale’s reliability. The Cronbach’s alpha for AMNPC and AMNKQ were 0.77 and 0.75, respectively, indicating a satisfactory internal consistency.

### Self-directed learning for the intervention group

The SDL model was conducted to teach the intervention group of working nurses the concept and competencies of AM. After the assessment of nurses’ readiness to utilize the SDL approach by Tool 1, Knowles’ SDL principles (1975) [22](Fig. 1) were implemented as follows: The participants engaged in a deliberation process to formulate and establish a prearranged course of action. This plan involved defining learning objectives, organizing outlines, arranging activities in a specific order, and setting a timeline for completing the activities within one month. In addition, the participants were provided with a diverse selection of EBP resources and learning materials. These resources were reviewed by nursing experts, and each participant chose the ones that aligned with their preferred learning styles and preferences. Examples of such resources include the Egyptian Knowledge Bank and the book library. The clinical instructor serves as a facilitator, providing regular feedback to both clinical instructors and peers. In addition, participants actively engaged in self-reflection about this learning experience.

### Traditional learning model for the control group

The control group of nurses learned the concept of AM and related nursing competencies using the regular clinical teaching approach familiar to the participants. A one-month plan was implemented during regular working hours. We divided the control group into two equal subgroups, each receiving pre-scheduled integrated lectures on the theoretical foundations of AM. There were a total of four lectures, each lasting two hours. The lectures were held once a week and participants received written handouts. The scientific content was adopted from different reviews of the literature [4, 6]. After each driven lecture, the researcher gave a supportive four-hour clinical application in a clinical setting utilizing the “see one-do one” clinical learning method. Furthermore, clinical supervision and guidance were given to the participants to ensure their ability to apply acquired knowledge in a real clinical setting.

### Procedures of data collection

After the implementation was completed, the follow-up and evaluation process began. This involved monitoring the occurrence of airway-related incidents reported by nurses in both the control and study-selected units on a daily basis for a continuous three-month period using (tool 4). Subsequently, the proficiency and methodologies of the nurses in the control group were assessed on two occasions. The initial assessment took place immediately after the completion of the implementation, while the second assessment occurred three months later to gauge the extent to which the education and training had been retained. This evaluation was conducted using tools 2 and 3. In contrast, self-evaluation was utilized among the study group using tools (2, 3), and it was observed and rated by someone else (peer colleague). Peer evaluations were conducted anonymously, cross-verified by multiple peers, and regular feedback sessions were held to ensure consistency and objectivity in the evaluations. Also, peer raters underwent comprehensive training, practice sessions, consensus meetings, and ongoing calibration to ensure consistency and reliability. The study group was also evaluated two times, similar to the control group. Finally, the researcher conducted a comparative analysis between the SDL results in the study group and the baseline data collected from the control group that underwent traditional ongoing learning practices.

### Data analysis

The study of both descriptive and inferential statistics was conducted using IBM SPSS Statistics version 23.0, IBM in New York. The data were presented using mean and standard deviation (SD). The data collected was assessed for normality using Shapiro-Wilk’s test and box plots. In order to examine the statistical differences between the control and intervention groups, an independent samples t-test was conducted with a significance level set at *P* < .05. Furthermore, the clinical significance was assessed using Cohen’s d effect size, where an effect size (ES) *≥* 0.4 was considered to be clinically meaningful [32]. In addition, an analysis of covariance (ANCOVA) was utilized to examine potential confounding factors that may have a statistical influence on the study’s dependent variable, with groups being considered as fixed factors.

### Result

**Fig. 2:**
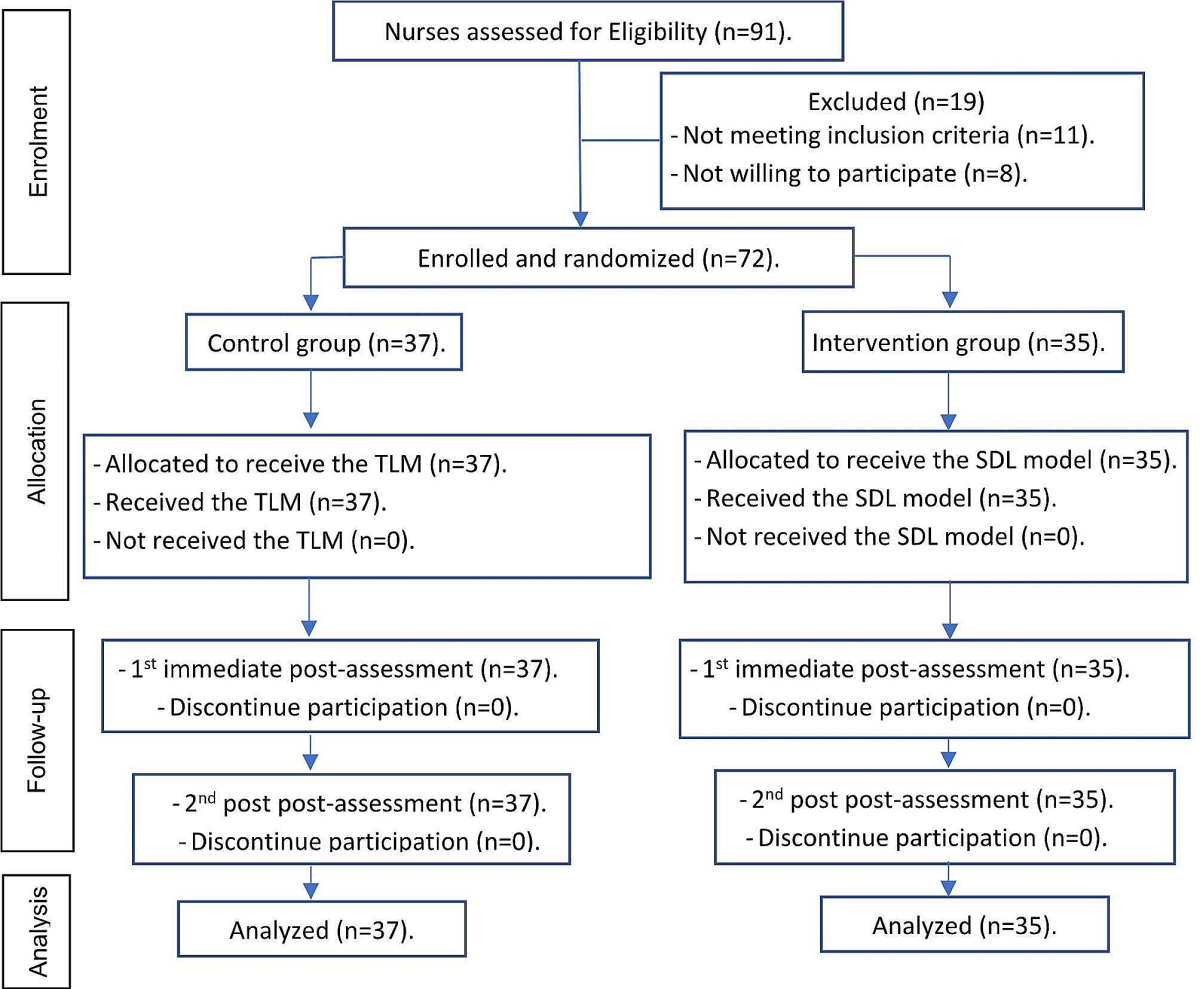
CONSORT flow chart shows subjects’ participation flow

A total of 91 nurses were assessed for their eligibility to partake in the present study. Out of these, 72 nurses were successfully enrolled and remained participants until the 2nd post-assessment, which marked the last stage of analysis, as reported in Fig. 2. The female participants accounted for 93% of the total, with 36.1% falling within the age range of 30 to 40 and 44.4% having accumulated 10 to 20 years of experience. In addition. 69.5% of the participants were diploma nurses. No significant difference was found between the intervention and control groups in terms of demographic features (Table 1).

**Table 1:** Demographic characteristics of the studied subjects (*n* = 72)

	Groups		
	Control (*n* = 37) *n* (%)	Intervention (*n* = 35) *n* (%)	*p*
**Gender**			0.597 ^a^
Male	2 (5)	3 (9)	
Female	35 (95)	32 (91)	
**Years of experience**			0.594 ^a^
1–10	11 (29.5)	9 (25.5)	
> 10–20	14 (38)	18 (51.5)	
> 20–30	9 (24.5)	7 (20.0)	
> 30–40	3 (8)	1 (3)	
Mean *±* SD	16.2 *±* 9.80	19.4 *±* 8.33	0.245 ^b^
**Educational level**			
Diploma nurses	26 (70.5)	24 (68.5)	0.868 ^a^
Technical nurses	8 (21.5)	9 (25.5)	
Bachelor’s nurses	3 (8)	2 (6.0)	
**Age**			0.757 ^a^
20–30	7 (19)	8 (23)	
>30–40	12 (32.5)	14 (40)	
>40–50	11 (29.5)	9 (25.5)	
>50–60	7 (19)	4 (11.5)	
Mean *±* SD	40.67 *±* 8.87	38.57 *±* 9.81	0.418 ^b^

^a^ Chi-square test

^b^ Independent Samples *t* Test

*SD* Standard deviation

A statistically significant difference was found between the control and intervention groups regarding their practice score during both the first and second points of assessments, with p-values of 0.01 and 0.02, respectively. The intervention group demonstrated the highest practice score (251.05 ± 12.37) during the initial assessment out of a maximum score of 300. Conversely, the control group had the lowest practice score (240.05 ± 10.36) during the subsequent assessment. In addition, the knowledge score was found to be significantly different between the control and intervention groups, either at the first or second assessment points, with p-values of 0.01 and < 0.01, respectively. The intervention group exhibited the highest scores in nurses’ knowledge during the initial assessment, with a mean score of 17.60 ± 0.94 out of a total of 20. The control group achieved the lowest knowledge score in the second assessment point, with a mean score of (13.62 ± 1.47). However, the overall nurses’ practices and knowledge scores were found to be statistically significantly different between the control and intervention groups with *p* < .01. Moreover, the difference found between control and intervention groups was clinically relevant, as evidenced by the effect size (ES) Cohen’s d in both practices and knowledge level (-0.56 and − 1.55, respectively) (Table 2). This result indicates significant differences between groups [31].

**Table 2:** A comparative analysis of knowledge and practice scores between intervention and control groups across two assessment points (*n* = 72)

	Max. score	Points of assessments	Groups		t	*p*	(ES) Cohen’s *d*
			Control (*n* = 37) Mean ± SD	intervention (*n* = 35) Mean ± SD			
**Practices** **level**	300	1st post-assessment	244.29 ± 9.36	251.05 ± 12.37	-2.60	0.01	-0.56*
		2nd post-assessment	240.05 ± 10.36	246.45 ± 12.90	-2.32	0.02	
		Total practices score	242.17 ± 9.81	248.75 ± 12.46	-2.495	< 0.01	
**Knowledge** **level**	20	1st post-assessment	14.75 ± 1.16	17.60 ± 0.94	-11.33	0.01	-1.55**
		2nd post-assessment	13.62 ± 1.47	16.42 ± 1.06	-9.19	< 0.01	
		Total knowledge score	14.18 ± (1.28)	17.01 ± (0.95)	-10.54	< 0.01	

*ES* Effect Size

* Medium effect size; **large effect size

*ES* *≥* 0.4 was considered clinically relevant

A significant difference was found between the immediate first post-assessment and the paired second (three months later) post-assessment concerning nurses’ knowledge and practices among control and intervention groups, as indicated by the paired t-test with *p* < .01 (Table 3). This difference occurs due to a significant decrease in the mean scores of nurses in the second assessment compared to the first assessment.

**Table 3:** Difference between the paired two times of assessment of nurses’ practices and knowledge within control and study groups

	Groups	Time 1 Mean ± SD	Time 2 Mean ± SD	Paired differences Mean ± SD	t	*p*
**Nurses’ practices**	Pair 1 (Control)	244.29 ± 9.36	240.05 ± 10.36	4.24 ± 2.15	11.90	< 0.01
	Pair 2 (Intervention)	251.05 ± 12.37	246.45 ± 12.90	4.60 ± 4.18	6.40	< 0.01
**Nurses’ knowledge**	Pair 1 (Control)	14.75 ± 1.16	13.62 ± 1.47	1.13 ± 0.71	9.67	< 0.01
	Pair 2 (Intervention)	17.60 ± 0.94	16.42 ± 1.06	1.17 ± 0.61	11.22	< 0.01

*SD* Standard deviation

The intervention group reported 18 airway-related incidents over three months, whereas the control group reported 24 incidents. Nevertheless, the observed difference was not statistically significant. The most prevalent type of airway-related incidents reported by both the control and intervention groups of nurses was airway injury, accounting for a total of 20 occurrences (47.6%). Conversely, the least frequent airway-related incident was aspiration, which was reported by both groups a total of seven times (16.6%) over a three-month period (Table 4).

**Table 4:** Differences in types and frequency of reported airway-related incidents as reported by the participants (*n* = 72)

Types	Control group (*n* = 37)		Intervention group (*n* = 35)		$\:{x}^{2}$	*p*
	Yes	No	Yes	No		
Airway obstruction	9	28	6	29	0.56	0.45
Airway injury	11	26	9	26	0.14	0.70
Aspiration	4	33	3	32	0.10	0.74

Covariance analysis was conducted to examine the relationship between nurses’ practice scores at the second point (dependent variable) and participants’ groups (fixed variable). The results showed that nurses’ practice scores at the first point were a significant confounding variable (f = 688.96, *p* < .01). After adjusting for covariates, such as nurses’ demographic characteristics, nurses’ knowledge, and airway-related incidents, there was no statistically significant impact on the nurses’ practice scores at the second point. In addition, the relationships between groups and study-dependent variables did not demonstrate a statistically significant effect, as indicated by low values of partial eta squared (< 0.02), as shown in Table 5. This finding suggests that the tested variables have a relatively minor impact on the dependent variable being examined.

**Table 5:** ANCOVA analysis for the nurses’ practices at the second point as a dependent variable (*n* = 72)

		*n*	Mean ± SD	SS	MS	F	*P*	η^2^
Intercept				39.75	39.75	3.54	0.06	0.05
Groups	Control Intervention	37 35	240.05 ± 10.36 246.45 ± 12.90	1.31	1.31	0.11	0.73	0.00
Gender	Male Female	5 67	243.85 *±* 13.89 243.09 *±* 11.92	12.25	12.25	1.09	0.30	0.01
Educational level	Diploma nurses Technical nurses Bachelor’s nurses	50 17 5	241.89 *±* 11.25 242.25 *±* 12.56 248.38 *±* 12.99	1.20	1.20	0.10	0.74	0.00
Age	20–30 > 30–40 > 40–50 > 50–60	15 26 20 11	245.72 *±* 12.27 240.52 *±* 11.15 246.22 *±* 13.46 241.07 *±* 10.57	0.11	0.11	0.01	0.92	0.00
Years of experience	1–10 > 10–20 > 20–30 > 30–40	20 32 16 4	246.25 *±* 16.86 242.28 *±* 9.23 239.68 *±* 10.20 248.75 *±* 6.39	9.03	9.03	0.80	0.37	0.01
Airway-related incidents				0.69	0.69	0.06	0.80	0.00
Nurses’ practice at 1st point				7726.33	7726.33	688.96	< 0.01	0.92
Nurses’ Knowledge at 2nd point				18.02	18.02	1.60	0.21	0.02
Groups * practice at 1st point				10.22	10.22	0.91	0.34	0.01
Groups * Knowledge at 2nd point				10.05	10.05	0.89	0.34	0.01
Groups * Airway-related incidence				0.14	0.14	0.01	0.91	0.00

R Squared=. 934; Adjusted R Squared = 0.922, SS Sum of squares, MS Mean square, *η*^2^ partial eta squared, SD Standard deviation

## Discussion

The current study is the first in Egypt to examine the impact of utilizing the SDL model compared to TLM among working nurses, focusing on enhancing their competencies regarding AM. The findings presented both clinical and statistical evidence to support the hypothesis that the self-directed clinical learning model enhances nurses’ practices and knowledge levels, leading to improved competency compared to the TLM. Moreover, the improvement was found at two different time points: the first was immediately after the implementation of the intervention, and the second was three months later, serving as a follow-up point.

Nurses may favor SDL due to its capacity to provide flexibility in terms of time and learning modalities. This adaptability can be particularly beneficial for working nurses with varying schedules and commitments. In addition, nurses may feel they take control of their learning process. Additionally, the nurses’ readiness to engage in SDL activity was emphasized as a prerequisite to participate in the study’s intervention group. Those nurses who exhibit readiness for SDL are more likely to demonstrate higher motivation levels to continue learning and enhance their capabilities [33]. The current study’s findings are consistent with the literature that states SDL is more effective than TLM in increasing nurses’ knowledge and practices [34, 35].

The knowledge and practice scores of the nurses in both intervention and control groups decreased over time, three months after the initial assessment following the educational content. A plausible explanation is that the duration for which acquired knowledge and skills are retained tends to decrease over time. Nevertheless, the lack of supervision and the heavy workload can also contribute to this result. This finding is consistent with previous research findings [36]. The difficulty in maintaining nurses’ competencies can be attributed to nurses’ attitudes, reluctance to change, lack of motivation, and inadequate dedication. This discovery aligns with prior research [37, 38].

The intervention group of nurses reported a lower incidence of airway-related incidents in their setting compared to the control group, based on the participants’ reports. Although the statistical significance was not observed, the results imply that the utilization of SDL may have a comparable impact to TLM in decreasing airway-related accidents, which serves as a reliable indicator of providing safe care. Furthermore, the minimal decrease in airway-related incidents may be attributed to a higher level of nurses’ knowledge and practices in the intervention group compared to the control group. Several researchers highlight the significance of training nurses as a highly effective approach to ensuring the maintenance of patients’ airways and the prevention of related complications [7, 21]. However, the occurrence of airway-related incidents is influenced by various factors beyond nurses’ competency, including patient-related aspects such as consciousness level, the accessibility of airway-related equipment and supplies, and the involvement of medical staff.

The reported airway-related incidents indicate that airway injury was the most frequently occurring type, while aspiration was the least common. This finding aligns with a previous prospective study that examined airway incidents over a period of six months [39]. Airway injuries are common among critically ill patients due to airway maintenance procedures such as suctioning, endotracheal intubation, and the use of airway patency equipment. Many other research has evidenced the high frequency of airway injuries [40, 41].

The current study implies that the nurses’ practice at the first point substantially influenced their practice scores at the second point. It suggests that interventions or improvements in practice may need to be targeted at an early stage to have a sustainable impact by keeping nurses motivated and emphasizing adequate supervision. Organizational commitment and support are essential, along with effective leadership, to keep nurses encouraged to continue refining their practices [42]. However, nurses’ demographic characteristics and knowledge level were not given significant emphasis as confounding variables in the nurses’ practice when the participants’ groups were considered fixed variables. The influence of SDL on nurses’ practice outcomes may be more significantly influenced by other prominent factors such as workload, motivation, learning style, and environmental factors. Furthermore, studies have demonstrated that problem-solving ability, self-efficacy, learning attitude, and learning interest were evidenced as factors affecting SDL implications; other studies suggest that collectively, both external and internal elements should be considered while implementing SDL [43, 44].

## Conclusion

In order to improve the quality of nursing care and maintain patients’ safety, the SDL model demonstrated a positive effect on nurses’ competencies in AM, as indicated by the improvement in their knowledge and practices compared to the TLM. However, the models of learning that the nurses used did not significantly affect the reported airway-related incidents. Therefore, to achieve a significant result in reducing airway-related incidents, it is necessary to manage and regulate various additional factors, including environmental conditions, staffing levels, equipment availability, and collaboration with other staff. Given the study’s findings that both the intervention and control groups experienced a decline in their knowledge and practices over time, it is crucial to examine the factors that hinder nurses from consistently applying what they have learned. It is necessary to identify an appropriate strategy, such as adequate supervision, to ensure the long-term sustainability of nurses’ performance. Furthermore, the observed improvement in nurses’ proficiency resulting from the SDL, as compared to the TLM, was not influenced by the nurses’ demographic characteristics. Consequently, the SDL approach can be beneficial for all nurses, irrespective of their differences. Finally, the investigators suggest refining nurses’ competencies using the SDL model, particularly for those who exhibit readiness. Besides, it is essential to establish collaborative learning environments with other team members to improve overall patient outcomes.

### Limitation

Given that the frequency of airway-related incidents in this study is solely based on participant reports, the primary concern is the possibility of underreporting bias. This bias stems from the potential reluctance of nurses to report or disclose airway-related incidents. The study was conducted in only one healthcare setting; therefore, examining the study variables in different settings is recommended to ensure generalization. The current study was an “open-label” study, as participants were informed about their group assignment (intervention or control), potentially impacting their behavior or responses. A blinding methodological approach is recommended in future research.

## Data Availability

The tools utilized for data collection, SDLRS, AMNKQ, AMNPC, and PSIR, in addition to the raw data of this study, are available from the corresponding author upon request.
